# Analysis of swallowing and voice-related quality of life in patients after supracricoid partial laryngectomy

**DOI:** 10.1007/s00405-023-08416-9

**Published:** 2024-01-06

**Authors:** Tianzhen Liu, Huajun Feng, Zhuoping Liang, Shengen Xu, Gang Qin

**Affiliations:** Department of Otolaryngology Head and Neck Surgery, The Affiliated Hospital of Southwest Medical University, Luzhou, 646000 China

**Keywords:** Laryngectomy, Swallowing, Voice, Dysphagia, Quality of life

## Abstract

**Objective:**

This study evaluated the swallowing and voice function of laryngeal cancer patients after Supracricoid Partial Laryngectomy(SCPL), and its influence on quality of life to provide a reference for the selection of surgical methods for laryngeal cancer patients.

**Methods:**

Twenty-one patients who received SCPL between April 2015 and November 2021 were included. Each patient’s swallowing function and quality of life were assessed through fiberoptic endoscopic examination of swallowing (FEES) and the M.D. Anderson Dysphagia Inventory (MDADI). Fundamental, jitter, shimmer, maximum phonation time (MPT), and voice handicap index-10 (VHI-10) were performed to assess voice function and voice-related quality of life.

**Results:**

The results of the FEES of the 21 patients were as follows: the rates of pharyngeal residue after swallowing solid, semiliquid, and liquid food were 0%, 28.57%, and 38.09%, respectively; the rates of laryngeal infiltration after swallowing solid, semiliquid, and liquid food were 0%, 28.57%, and 4.76%, respectively; and aspiration did not occur in any of the patients. In the evaluation of swallowing quality of life, the mean total MDADI score was 92.6 ± 6.32. The voice function evaluation showed that the mean F0, jitter, shimmer, and MPT values were 156.01 ± 120.87 (HZ), 11.57 ± 6.21 (%), 35.37 ± 14.16 (%) and 7.85 ± 6.08 (s), respectively. The mean total VHI-10 score was 7.14 ± 4.84.

**Conclusion:**

SCPL provides patients with satisfactory swallowing and voice function. The patients in this study were satisfied with their quality of life in terms of swallowing and voice. SCPL can be used as a surgical method to preserve laryngeal function in patients with laryngeal cancer.

## Introduction

Supracricoid partial laryngectomy was originally proposed by Majer et al. as an operative method that preserves laryngeal function in individuals with laryngeal cancer and was later modified by Piquet et al. [1, 2]. SCPL can be used in the treatment of early- and advanced-stage laryngeal cancer or in laryngeal cancer patients for whom radiotherapy has failed [3, 4]. Compared with a total laryngectomy, SCPL can achieve a good local control rate [5] and preserve patients' swallowing, breathing, and articulation functions.

SCPL requires the retention of the cricoid cartilage, at least one arytenoid cartilage, and the mobile cricoarytenoid joint. Depending on the scope of resection and the anastomosis, the surgical methods can be divided into cricohyoidopexy (CHP), cricohyoidoepiglottopexy (CHEP), tracheocricohyoidopexy (TCHP), and tracheocricohyoidoepiglottopexy (TCHEP). Due to the absence of most of the laryngeal cavity structure in postoperative patients, swallowing dysfunction and voice dysfunction are common postoperative complications.

In the short term, all patients with SCPL will have a certain degree of dietary choking cough that may lead to aspiration pneumonia and can be life-threatening in severe cases. However, different research institutions have reached different conclusions regarding whether patients who undergo SCPL have chronic aspiration more than half a year after surgery, whether the quality of life of these patients is affected by aspiration, and whether total laryngectomy is required in individuals with serious and persistent aspiration [6–12].

After bilateral excision of the vocal cords in SCPL, postoperative voice articulation relies mainly on the newly formed glottic structure, which includes the arytenoid cartilage mucosa, the tongue root mucosa, the piriform fossa mucosa, and the epiglottic mucosa [13, 14], and postoperative hoarse voice is inevitable. Commonly used indicators for the evaluation of voice function include jitter, shimmer, F0, and MPT [15]. F0 reflects the basic frequency of vocal chord vibration, shimmer and jitter reflect the hoarseness and the roughness of the patient's voice, respectively, and MPT is an important indicator of laryngeal aerodynamics. At present, there are few studies on the recovery of voice function and quality of life in patients after SCPL [16, 17].

Therefore, this study evaluated postoperative swallowing, voice function, and quality of life in patients who underwent SCPL in our hospital between April 2015 and November 2021. To provide a basis for the selection of SCPL as a surgical method for the clinical treatment of laryngeal cancer patients.

## Materials and methods

### Patients

Clinical data of patients who underwent SCPL in the Department of Otolaryngology-Head and Neck Surgery of the Affiliated Hospital of Southwest Medical University between April 2015 and November 2021 were analysed. Information on nasogastric tube and tracheal tube removal, postoperative radiotherapy, postoperative complications, recurrence and metastasis, and treatment results was recorded and analysed. TNM staging was performed according to the 7th edition of the Staging Guidelines of the Union for International Cancer Control (UICC).

Swallowing and voice-related quality of life after SCPL were analysed in all enrolled patients except those to whom any of the following conditions applied: (1) patients for whom less than half a year had elapsed after surgery; (2) patients who were lost to follow-up or died; (3) patients in whom the mandible, tongue, soft palate, oesophagus, or other structures were removed prior to the swallowing function evaluation, since loss of these structures may affect recovery of swallowing function; (4) patients who refused to participate in a functional assessment or had serious consciousness disorders that rendered them unable to cooperate with the examination. For the patients included in the functional assessment, recovery of swallowing, voice function, and quality of life were assessed during outpatient follow-up.

### Treatment plan

All patients were operated on by the same experienced head and neck surgery team. The scope of the tumour lesion and its relationship to the adjacent tissues were investigated by preoperative fiberoptic laryngoscopy, neck-enhanced CT, and neck-enhanced MRI. CHP surgery is used in cases with supraglottic laryngeal cancer, CHEP surgery is mainly used in cases with glottic laryngeal cancer, and TCHEP surgery is used if the tumour involves the area below the glottis and near the cricoid cartilage arch. Whether cervical lymph node dissection was performed was determined according to the cervical lymph node metastasis shown by cervical enhanced CT and MRI scans and the rule of lymph node metastasis at the location of the primary tumour. Whether to administer supplemental radiotherapy after surgery was determined comprehensively according to the operative margin, the primary tumour range, and the degree of cervical lymph node metastasis.

### Swallowing function

Swallowing function was assessed using a fiberoptic laryngoscope (VNL-1570STK) connected to a video recorder (LMD-1951MC). The end of the laryngoscope was inserted through the nasal cavity on one side and placed between the soft palate and the epiglottis to permit observation of the entire root of the tongue and the entire laryngopharyngeal cavity. Fluid or food retention in the patient's epiglottic valley and piriform fossa before swallowing was observed. The subject was asked to swallow emotively three times if there was retention. The subjects ate 10 g of black biscuits, 10 ml of yogurt, and 10 ml of water containing blue dye, and whether there was retention of fluid or food in the epiglottic valley and piriform fossa was observed. If retention occurred, the subject was asked to swallow three times, and food retention was again evaluated. Pharyngeal residue was assessed using the Yale Pharyngeal Residue Severity Rating Scale [18, 19]. Food aspiration was assessed using the penetration-aspiration scale [20].

### Voice function

Voice function assessment was performed using on-site instruction by 3 medical professionals who specialize in voice. To perform an acoustic analysis of the patient’s voice, the patient’s voice was transmitted through a microphone (centre 320) and a USB sound card (UCA222) and recorded and analysed by LingWaves software (version 3.2.3). The ambient noise was less than 40 dB, and the microphone was placed 30 cm from the lips. The patient was instructed to continuously pronounce the /a/ sound, and the test was repeated 3 times. The most stable sound samples obtained from each patient were used to determine the basic acoustic indicators of voice: F0, jitter, and shimmer. The voice dynamics index MPT was measured by asking the patient to continuously pronounce the vowel /a:/ after three or more deep inspirations. The longest and most stable sound sample was selected and recorded as the MPT.

### Swallowing and voice-related quality of life assessment

Swallowing quality of life assessment was performed using the MDADI [21, 22], which was completed by the patient under the supervision of a fixed researcher. The MDADI is a questionnaire on quality of life for patients with head and neck cancer; it includes a total of 20 questions divided into 4 subcategories: general (1 question), emotional (6 questions), functional (5 questions), and physiological (8 questions). A Likert 5-level scoring method was adopted; the responses “fully agree”, “agree”, “do not know”, “disagree”, and “completely disagree” were scored as 1, 2, 3, 4 or 5 points, respectively, and the total score was 20–100 points. A total score of less than 60 is defined as swallowing dysfuntion [23]. The higher the score, the better the patients rated their quality of life in terms of swallowing.

Voice quality of life was assessed using the VHI-10 questionnaire [24, 25]. This questionnaire was completed by the patients themselves under the guidance of a single researcher. The VHI-10 contains 10 questions that are divided into 3 subcategories: function (5 questions), physiology (3 questions), and emotion (2 questions). A 5-point Likert scale scoring method is adopted: the answers “none”, “rarely”, “sometimes”, “often”, and “always” are assigned 0, 1, 2, 3 and 4 points, respectively. The lowest possible score is 0, and the highest possible score is 40. An overall score greater than 10 is defined as voice dysfuntion [26]. The lower the score, the less the patients thought the voice disorder affected their quality of life.

### Statistical analysis

Spearman correlation analysis was used to analyse the factors affecting patients' voice function. SPSS software (version 22.0) was used to perform data analysis. *P* < 0.05 was considered statistically significant.

## Results

### Clinical characteristics of the patients

All patients were male. The ages of the 21 patients ranged from 44 to 69 years, with a median of 55 years. The follow-up time was 6 to 78 months, and the median follow-up time was 29 months. Of the 21 patients, 7 underwent bilateral neck lymph node selective dissection, and 3 patients were confirmed to have lymph node metastasis by postoperative pathology. Bilateral arytenoid cartilage was preserved in all patients. Six patients received postoperative radiotherapy, with an average dose of 63.8 ± 4.3 Gy (56–75 Gy). Other clinical characteristics of the patients are shown in Table 1.

**Table 1:** Demographic and clinical characteristics of patients

Clinical characteristics	Number of cases (n)	Rate (%)
Age (years)		
≥ 60	5	23.81
< 60	16	76.19
Smoking history		
No	1	4.76
Yes	20	95.24
Differentiation grade		
Poor	1	4.76
Middle	5	23.81
Well	15	71.43
Tumour site		
Glottic	20	95.24
Supraglottic	1	4.76
T classification		
T1b	10	47.62
T2	4	19.05
T3	7	33.33
N classification		
N0	18	85.72
N1	2	9.51
N2	1	4.77
Clinical stage		
I	10	47.62
II	4	19.05
III	6	28.57
IVa	1	4.76
Type of operation		
CHP	1	4.77
CHEP	18	85.72
T-CHEP	2	9.51

### Postoperative rehabilitation

All patients had gastric tube extubation. The tracheal catheter was extracted in 18 cases, corresponding to an extubation rate of 85.72%. 1 patient developed pulmonary infection after the operation, and was cured after anti-infection treatment. Infection at the incision site occurred in 4 patients, and all healed after dressing changes and anti-infection treatment. Postoperative laryngeal stenosis occurred in 3 cases, and 1 patient underwent laryngoplasty for laryngeal stenosis.

### Postoperative recurrence and metastasis

Postoperative metastases were observed in 1 patient: thyroid metastasis. The patient underwent total thyroidectomy, central lymph node dissection, and bilateral cervical lymph node selective dissection. No patients experienced recurrence after surgery.

### Swallowing function

**Fig. 1:**
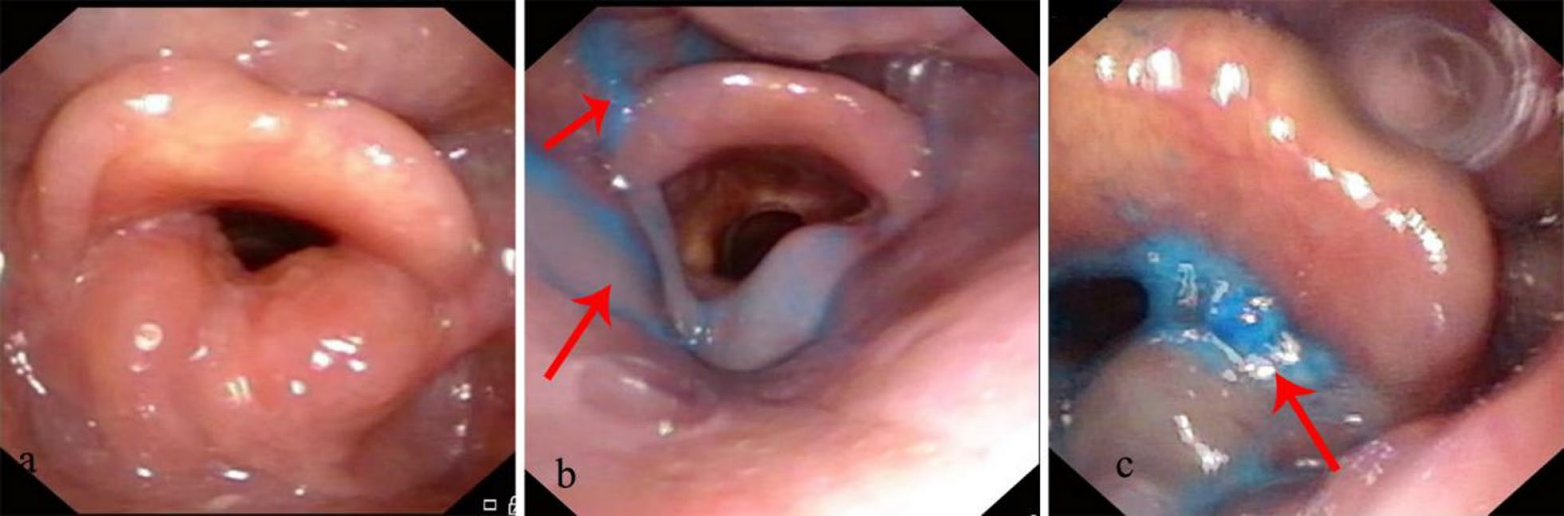
**a** Normal swallowing function; **b** Pharyngeal residue; **c** Laryngeal penetration

The results of the fiberoptic laryngoscope swallowing function evaluation of 21 subjects are presented in Table 2. Regarding pharyngeal residue (Table 2 and Fig. 1a), the residual rate after ingestion of solid food was 0%, that after ingestion of semiliquid food was 28.57%, and that after ingestion of liquid food was 38.09%. The results regarding aspiration are shown in Table 3 and Fig. 1c. There was no case of aspiration after swallowing of solid, semiliquid, or liquid food; thus, the incidence of aspiration was 0%. The laryngeal infiltration rate after ingestion of solid food was 0%, that after ingestion of semiliquid food was 28.57% and that after ingestion of liquid food was 4.76%.

**Table 2:** Pharyngeal residue (n = 21)

Groups	Solid	Semiliquid	Liquid
With residue	0	6	8
Without residue	21	15	13

**Table 3:** Laryngeal infiltration (n = 21)

Groups	Normal	Laryngeal penetration	Aspiration	Static aspiration
Solid	21	0	0	0
Semiliquid	15	6	0	0
Liquid	20	1	0	0

### Voice function

In 21 patients, the mean values of each index of voice acoustic analysis were as follows: jitter, 11.57 ± 6.21 (%); shimmer, 35.37 ± 14.16 (%); and F0, 156.01 ± 120.87 (Hz). The aerodynamic index indicated a mean maximum phonation time of 7.85 ± 6.08 (s) (Table 4). The Spearman test for correlations among the postoperative voice acoustic analysis results, aerodynamic indices, and clinical factors found that MPT was positively correlated with postoperative time; the Spearman correlation coefficient was 0.453 (*P* = 0.039) (Table 5).

**Table 4:** Spearman test for correlations among postoperative voice function indices and clinical data

Groups	Age		Postoperative time		Postoperative radiation		T classification	
	*r*	*P*	*r*	*P*	*r*	*P*	*r*	*P*
F0	− 0.352	0.117	− 0.382	0.088	0.000	1.000	− 0.074	0.750
Jitter	− 0.130	0.575	− 0.239	0.296	0.122	0.599	− 0.160	0.489
Shimmer	− 0.168	0.466	− 0.151	0.513	− 0.052	0.822	− 0.023	0.920
MPT	0.193	0.401	0.453*	0.039	− 0.070	0.762	0.072	0.757

*r* Spearman correlation coefficient

*Statistically significant, *P* < 0.05

**Table 5:** MDADI and VHI-10 scores of different groups (mean ± SD) (n = 21)

Groups	MDADI	VHI-10
Total	92.67 ± 6.32	7.14 ± 4.84
Functional	20.67 ± 1.49	4.14 ± 3.08
Physical	38.48 ± 2.70	2.57 ± 1.66
Emotional	29.24 ± 1.90	0.42 ± 0.97

### Swallowing and Voice-related quality of life

The mean total MDADI score of 21 subjects was 92.67 ± 6.32, much higher than 60, indicating that the patients were satisfied with their own quality of life in swallowing. The mean total score of 21 patients on the VHI-10 was 7.14 ± 4.84, lower than 10, indicating that patients were satisfied with their own voice quality of life.

## Discussion

As one method of partial laryngectomy, SCPL can preserve the patient's breathing, swallowing, and articulation functions while accomplishing removal of the lesion and ensuring a safe incision margin. Allegra et al. analysed the survival of 72 patients with glottic laryngeal cancer after CHEP surgery and concluded that disease-specific survival and local relapse-free survival after this procedure were both greater than 90% [27]. Pescetto et al. analysed the survival of patients who had received SCPL and found that 5-year overall survival, disease-specific survival, and disease-free survival were all greater than 80% and that the local control rate reached more than 90% [28]. More research has found that SCPL results in a higher postoperative survival rate and a higher local control rate, and that it produces better oncological effects and quality of life [29–31]. In clinical work, physicians should select appropriate treatment methods for patients based on factors such as tumour site, the patient's physical condition, and their own judgement.

SCPL removes most of the laryngeal structure, leading to the risk of transient swallowing coughing and even aspiration. Simonelli et al. used FEES to determine the swallowing recovery of 116 patients at least 3 years after SCPL and showed that 79 (68.1%) of those patients had aspiration, but no obvious pulmonary inflammation was observed on high-resolution chest CT [7]. Di Santo et al. retrospectively analysed the swallowing function of 39 patients at least 3 years after SCPL through FEES and found that 28 (71.8%) had pharyngeal retention and 14 (35.9%) had aspiration, but no patient underwent total laryngectomy due to severe aspiration [6]. In this study, no food residue or laryngeal infiltration occurred when the patients swallowed solid food after SCPL; the pharyngeal residue rate and the laryngeal infiltration rate when swallowing semiliquid and liquid food were both low, and no patient had aspiration when swallowing any of the three types of food. This may be related to the fact that most of the patients in this group were evaluated at least half a year after surgery, and all of them had actively participated in swallowing training after surgery.

The patients in our study who had stage T1b tumours had their gastric tubes removed successfully after surgery, and their swallowing function recovered well. Despite the occurrence of food residue and laryngeal infiltration, there was no case of aspiration. Therefore, the selected patients with early laryngeal cancer treated with SCPL also had good swallowing function. For patients with early glottic laryngeal cancer who refuse radiotherapy or whose tumours cannot be fully exposed under an endoscope, SCPL can be used as an effective surgical method for preservation of open laryngeal function. At the same time, in laryngeal cancer patients who meet the indications for SCPL, swallowing function can be recovered well by participation in strengthening swallowing training after surgery.

The MDADI scale is commonly used to evaluate swallowing function after surgery in patients with head and neck tumours [21, 32]. The scale evaluates three aspects: social life, psychological state, and functional recovery. The higher the score on the scale, the more satisfied patients are with their swallowing-related quality of life. Topaloğlu et al. and Schindler et al. found that the total MDADI score of patients who had received SCPL was more than 80 points [24, 33]; the mean total MDADI score in this group was 92.67 ± 6.32, indicating that patients were satisfied with their postoperative swallowing quality of life. Recovery of swallowing function and swallowing quality of life after SCPL can be affected by several factors, including arytenoid cartilage resection, preoperative and postoperative radiotherapy, and age. Topaloğlu et al. and DiSanto et al. found that material retention was significantly increased in patients who received radiotherapy, but no significant difference in MDADI score was found [6, 33]. At the same time, these data also showed that although semiliquid food residue and laryngeal infiltration were affected by postoperative radiotherapy, in the instrument evaluation it was found that patients' swallowing quality of life was not affected by age or postoperative radiotherapy. The above studies show that postoperative swallowing function recovery may be affected in patients who receive postoperative radiotherapy and arytenoid cartilage resection, but patients are more satisfied with their own swallowing quality of life.

After SCPL, patients mainly rely on neolaryngeal articulation. Compared with the normal vocal cords, the quality of the newly formed glottis structure after surgery is increased, and the types of vibrating sound sources are increased, leading to roughness and hoarseness in the patients’ voices. F0 reflects the frequency of vocal cord vibration, jitter reflects the periodic change in adjacent sound waves, shimmer reflects the degree of change in the amplitude of adjacent sound waves, and MPT reflects the ability of the larynx to produce a continuous sound. In previous studies that used objective indicators of the voice function of patients after SCPL and that of a control group, it was found that F0 and MPT decreased and shimmer and jitter increased; this objectively reflected that the postoperative pitch of the patients’ voices decreased, the roughness and hoarseness of their voices increased, and the maximum phonation time decreased. The fluctuation ranges of each indicator were as follows: F0, 70.1–154 (Hz); jitter, 3.4%-10.9%; shimmer, 2.2–25%; and MPT, 4.1–11.3 (s) [15, 16, 34–36]. The mean values obtained for jitter (11.57 ± 6.21 (%)), shimmer (35.37 ± 14.16 (%), F0 (156.01 ± 120.87 (Hz)) and MPT (7.85 ± 6.08 (s)) in our study were close to the above means. In addition, no correlations were found among F0, jitter, shimmer, postoperative radiotherapy, postoperative time, T stage, or age in this study, consistent with the results obtained by Makeieff et al. [27], suggesting that the roughness and hoarseness of patients' voices are affected by various factors and that more clinical studies are needed. On the other hand, this study found that MPT was positively correlated with postoperative time, indicating that MPT increased gradually with the extension of postoperative time. In a study of voice changes in 17 patients with SCPL-CHEP 1 year, 1–3 years, and 3 years or more after surgery, Miyamaru et al. found that the mean value of MPT increased gradually over the time periods [13]. This may be related to the gradual extension of the postoperative time, a gradual increase in the vocal adaptability of the surgically modified larynx, and improvement in the closure of the new glottis.

For the investigation of patients’ voice-related quality of life after SCPL surgery, the VHI-10 scale, which includes the impact of a voice disorder on the patient’s social life, psychological state, and pronunciation status, is commonly used. The lower the total score on this scale is, the smaller the impact of a voice disorder on the patient’s quality of life. In this study, the mean total VHI-10 score of the patients was 7.14 ± 4.84, indicating that patients were satisfied with their voices and that they believed that the voice disorder did not have a serious impact on their quality of life. This is consistent with the study of Miyamaru et al. [13, 15]. In general, the patients were satisfied with their voice quality of life, even though their new glottic closure ability and vocal ability were poor after SCPL and the degree of hoarseness and roughness of the voice was significantly increased. The results were not surprising, as the patients had been prepared for the removal of their vocal cords before surgery. In addition, with the extension of postoperative time, patients have gradually adapted to the sound generated by the new glottic structure, and after surgery, they only meet the requirements of daily life communication, except for patients who need to continue to work.

## Conclusion

This study evaluated recovery of swallowing, voice function, and voice quality of life in laryngeal cancer patients after SCPL using specific scales. It was concluded that the patients' swallowing and voice function recovered well and that the patients were satisfied with their quality of life. SCPL can be used as a surgical method to preserve laryngeal function in early- and advanced-stage laryngeal cancer.

## Data Availability

The data that support this study are available from the corresponding author upon reasonable request.
